# Steatotic Liver Disease in Allogeneic Hematopoietic Stem Cell Transplant Recipients: A Case Series and Literature Review

**DOI:** 10.3390/hematolrep18050065

**Published:** 2026-09-10

**Authors:** Karthik Chetlapalli, Clifford Shin, Stuart Seropian, Francine Foss, Iris Isufi, Sarah Perreault, Manoj Pillai, Amer Zeidan, Mahan Mathur, Noffar Bar, Charles Kenworthy, Gary Israel, Lohith Gowda

**Affiliations:** 1 Section of Hematology, Department of Medicine, Yale School of Medicine, New Haven, CT 06510, USAlohith.gowda@yale.edu (L.G.); 2 Department of Radiology & Biomedical Imaging, Yale School of Medicine, New Haven, CT 06510, USA

**Keywords:** steatotic liver disease, MASLD, hematopoetic stem cell transplantation, hematological malignancy, graft-versus-host-disease

## Abstract

**Background/Objectives**: Obesity and inflammatory conditions, including steatotic liver disease, are known to impact the hematopoietic niche and immune surveillance. Therefore, assessing the impact of steatotic liver disease on recipients of allogeneic stem cell transplantation (allo-HSCT) with bone marrow origin neoplasms is clinically relevant and an underexplored area of investigation. **Methods**: We followed the clinical course of allo-HSCT recipient patients with steatotic liver. **Results**: From 2014 to 2020 at our center, we identified 18 patients (5.8% of 306 patients screened) with steatotic liver disease detected on non-contrast CT imaging pre-transplant. With a minimum of 5 years follow-up for all, eight patients experienced post-transplant relapses (44%). Relapses (78%) followed by infections (55%) were the major contributors of mortality in this cohort. Pre-transplant transaminases were normal (AST median 28, ALT median 37) in all, while most patients (89%; 16/18) developed abnormal transaminases in the first-year post-transplantation without evidence of permanent liver injury. None experienced veno-occlusive disease of the liver. The cumulative incidence of acute graft-versus-host disease (aGVHD) was 33% (6/18), with 55% (10/18) experiencing chronic graft-versus-host disease (cGVHD). **Conclusions**: Our descriptive study highlights that radiologically detected steatotic liver disease is not a contraindication to proceeding with allogeneic stem cell transplant, and its association with transaminitis, relapse, immune complications, and post-transplant metabolic health requires future mechanistic studies.

## 1. Introduction

With the rates of obesity and metabolic syndrome continuing to rise, there has also been an increase in the incidence of steatotic liver disease (SLD) and metabolic dysfunction-associated steatotic liver disease (MASLD) in the general population [1,2]. Steatotic liver disease is characterized by the pathologic accumulation of lipids within hepatocytes detected via imaging or biopsy, with MASLD defined as a subtype of SLD in the presence of cardio-metabolic factors [3]. Recent meta-analyses have estimated the global prevalence of MASLD at 38.0%, marking it as the most common chronic liver disease worldwide [1,4,5]. Furthermore, recent projections expect the prevalence of MASLD to rise toward 55.7% by 2040 [6,7]. Notably, accumulating evidence indicates disruption of normal immune function in patients with steatotic liver disease, ranging from increased infection risk and elevated inflammatory cytokines to impaired lymphocyte and monocyte function [8,9,10]. As a result, researchers are now investigating the mechanistic role of steatotic liver disease in disrupting the liver’s central role in immune surveillance and immunomodulation, thereby influencing malignancy development, autoimmune disorders, treatment resistance, and overall outcomes across different therapies [11,12].

Concurrently, allogeneic hematopoietic stem cell transplantation (allo-HSCT) is a potentially curative procedure for many hematological malignancies that otherwise carry a poor prognosis. Despite its potential benefits, several complications associated with this procedure, including graft-versus-host disease, infection, and veno-occlusive disease, can impact morbidity and mortality in those who seek a cure [13,14]. Most drugs used peri-allo-HSCT are metabolized in the liver, and hence optimal liver function is highly desirable for transplant recipients. During the post-transplantation period, preserving immune vigilance, which may decrease relapse risk post-transplant, and dampening the adverse allogeneic immune responses associated with graft-versus-host disease (GVHD) remain areas of ongoing research. Recently, Maung et al. [15] emphasized a potential link between steatotic liver disease and chronic graft-versus-host disease (cGVHD), but these findings have yet to be replicated in other large-scale studies.

Therefore, with limited available evidence to guide the management of allo-HSCT recipients with notable steatotic liver disease, our case series aimed to explore the perceived risks for such individuals pursuing curative-intent cellular immunotherapy and we report our findings here.

## 2. Materials and Methods

### 2.1. Patient Selection

Our cohort included patients who received allo-HSCT at Yale New Haven Hospital for hematological malignancy between 2014 and 2020 and had screening pre-transplant non-contrast CT scans performed. Independent radiologists then reviewed each of these patients’ imaging to detect steatotic liver disease, using the standardized CT criteria of at least a 10 Hounsfield unit difference in attenuation between the liver and the spleen [16]. The study was approved by an institutional review board.

### 2.2. Patient Characteristics and Methods

For each patient identified to have SLD, baseline disease status, treatment, and relevant clinical parameters were collected. Clinical parameters of interest were age at transplant, disease type, body mass index (BMI), and peri-transplant liver function test results. Steatosis risk factors and conditions like diabetes, hyperlipidemia, cardiac disease, hypertension, and alcohol abuse are often comorbid and were therefore also included when available. Pre-transplant hepatic injury or dysfunction was determined if AST, ALT, or bilirubin levels were above 1.5 times the upper limit of normal (ULN) of the local laboratory reference. Post-transplant AST, ALT, and bilirubin abnormalities had to reach a higher threshold of 2 times the ULN of the local laboratory reference to be considered abnormal. Each patient’s disease subclass, stem cell transplant conditioning regimens, and GVHD prophylaxis were also collected.

### 2.3. Mortality, Relapse, and Graft Versus Host Disease

Morphologic relapse, GVHD, and mortality were key outcomes identified and classified during the post-transplantation period. Patients were followed from the date of allogeneic hematopoietic stem cell transplantation until death or last-known follow-up. The data cutoff was April 2026. Mortality was further subclassified by causes of death listed on death certificates and/or most recent clinical documentation. The occurrence and severity of GVHD were also assessed from clinician notes and confirmed according to criteria set forth by the NIH’s Consensus Development Project for chronic GVHD [17].

## 3. Results

### 3.1. Patient Characteristics

A total of 18 allo-HCST recipients (5.8%) met criteria for steatotic liver disease based on non-contrast CT scans performed before transplantation. Of these patients with radiologically confirmed steatotic liver disease, 11 were female, and seven were male. The median age at transplantation was 56 years (range 30–79 years). The primary indication for transplant included high-risk hematologic neoplasms like acute myeloid leukemia (44%; n = 8), acute lymphoblastic leukemia (27%; n = 5), and T-cell lymphoma (11%; n = 2). Notably, 83% (n = 15) had BMIs above 25, 22% (n = 4) had type 2 diabetes, and no patients reported a prior history of alcohol abuse disorder. Other pre-transplant comorbidities included hypertension (44%; n = 8) and hyperlipidemia (44%; n = 8). All other pre-transplant characteristics are summarized in Table 1.

### 3.2. Clinical Outcomes

Table 2 provides patient-level data on the primary outcomes of relapse, GVHD, and mortality. At a median follow-up of 90.5 months (range 67.6–132.4) among survivors, 9 of 18 patients had died. Relapses were the most common cause of treatment failure, occurring in 44% of patients (8 out of 18). Of those who relapsed, 75% (6 out of 8) experienced it within the first-year post-transplantation, with a median time to relapse being 6.8 months (range 0.4–16.7). Relapse also remained the most common cause of death (78%; 7 out of 9). Infection was reported as a contributing cause of death in 55% of patients (5 out of 9).

The cumulative incidence of all grades of acute GVHD was 33%, while the cumulative incidence of chronic GVHD in this study was 55%. Per the NIH cGVHD consensus criteria, there were 1 mild case, 5 moderate cases, and 4 severe cases. In these cases of cGVHD, a broad range of organ systems were affected, including the liver, GI system, lungs, fascia, skin, oral mucosa, and eyes.

Per the study definition, hepatic dysfunction or injury was observed in 22% of patients (4 out of 18) pre-transplantation, rising to 89% of patients (16 out of 18) in the first year post-transplant. No patient experienced veno-occlusive disease. Notably, only 4 patients met the criteria for hepatic cGVHD based on biochemical abnormality (>Score 0 hepatic GVHD per NIH criteria), but none of the deaths were attributed to hepatic GVHD. The remaining 12 patients did not meet the criteria for hepatic GVHD outlined in Jagasia et al. but were more closely monitored for hepatic function amongst other workup [17].

## 4. Discussion

Preserved multiorgan function is critical to the overall success of allo-HSCT. Among the body’s organs, the liver plays a central role in controlling drug metabolism and fundamental cellular responses (metabolic, inflammatory, immune, etc.) [18,19,20,21]. In the context of allo-HSCT, teasing apart these multifaceted liver processes is often challenging, and pursuing a biopsy of the liver for diagnostic reasons can be a high-risk intervention. Consequently, radiologic evaluation combined with biochemical profiling of hepatocellular injury and synthetic function often provides a preliminary non-invasive means to assess pre-transplant hepatic health and potential post-transplant clinical outcomes. Historically, obesity (reflected by BMI) and post-transplant hyperglycemia, as surrogate markers for metabolic derangements, have been linked to higher mortality and morbidity rates among HSCT recipients, particularly due to infections and GVHD [11,22,23,24]. Several other reports suggest that obesity, a risk factor for MASLD, influences the success of immunotherapies, with paradoxical findings observed across cancer types [9,10,12,25,26].

For these reasons, our study explored the significance of subclinical steatotic liver disease detected by radiologic tests during the pre-transplant screening period and reported clinical events post-allo-HSCT in this perceived high-risk cohort.

### 4.1. Relapse Risk

Overall, our study’s cumulative incidence of relapse was 44%, which approximates the real-world relapse risk for allo-HSCT recipients with AML (44% of our cohort) [27,28,29]. Importantly, as a single-cohort descriptive study, relative relapse risk could not be assessed due to diversity in preparative regimen intensity, disease histology, and GVHD prophylaxis. Nonetheless, as a neutral signal, our results do fit into a growing body of literature. A prior study evaluating the impact of pre-transplant SLD similarly did not see an elevated rate of relapse or mortality (both relapse and non-relapse) [15]. Conversely, across organ systems affected by fatty deposition, preclinical studies have shown that fatty deposition can alter the hematopoietic milieu and thymic (T cell maturation), which are essential for immune surveillance. Whether this translates to human studies is an area of unmet research need [30]. Separately, post-transplant, treatment-associated steatotic liver disease has been noted across numerous centers, though its impact on relapse risk is still not clear [31,32].

### 4.2. GVHD Risk

Post-transplant GVHD can decrease quality of life and potentially decrease survival for patients after allo-HSCT. We found that 33% of our cohort experienced aGVHD (any grade), and 55% experienced cGVHD (any grade). Across reports in this space, Maung et al. from Duke University showed heightened chronic GVHD risk with pre-transplant steatosis, and others have shown post-transplant hyperglycemia as a potential risk factor [15,33]. Metabolic profiling, in conjunction with microbiota-derived products, has also been shown to control T cell alloreactivity in transplant recipients [34]. Finally, preclinical experiments have clearly shown that gluconeogenesis and glycolysis overwhelmingly determine T cell fate in GVHD models [35,36]. Based on these developments, we hypothesize that steatotic liver disease could be a potential risk factor for GVHD and that this effect is regulated by glucose levels, given the liver’s metabolic function, and that this effect needs further clinical validation.

### 4.3. VOD Risk

Our study did not identify any patients with veno-occlusive disease (VOD). Clinically, prior liver disease has been acknowledged as a risk factor for the occurrence of VOD; however, no study has established whether MASLD is a sufficient risk factor [32]. Theoretically, liver sinusoidal endothelial cell dysfunction has been noted in steatotic liver disease and therefore could be a factor in the development of veno-occlusive disease secondary to transplant conditioning [37]. Ultimately, further clinical studies are warranted.

### 4.4. Laboratory LFT Abnormalities

In assessing mortality and morbidity risk in transplant recipients, the Hematopoietic Cell Transplantation-specific Comorbidity Index (HCT-CI) is a validated tool that incorporates hepatic dysfunction as measured by numerical aberrations in bilirubin and transaminases [38]. However, these scoring models neither consider the etiology of longitudinal LFT abnormalities nor explain the mechanism for elevated relapse- and non-relapse-related mortality [39]. Similarly, within the grading of acute hepatic GVHD post-transplant, there is over-reliance on serum bilirubin levels, which can fluctuate from blood transfusions, drug injections, nutritional status, and enzymatic deficiencies in bilirubin metabolism [40]. Simultaneously, veno-occlusive disease of the liver and hepatic GVHD remain clinical suspicion-based diagnoses that require rapid intervention. Chronic use of interventions like steroids, though, can worsen steatosis, further clouding the clinical picture. Additionally, pathological examination of the liver from patients who succumbed to complications has shown mixed utility, with a plethora of observations documented [41,42]. Ultimately, as a potential modifier within this framework, the role of pre-existing steatosis in allo-HSCT patients remains largely unknown, and the utility of tracking liver function tests warrants further investigation.

In our study, 22% of patients pre-transplant and 89% post-transplant, when longitudinally monitored in the first-year post-transplant, met the criteria for hepatic dysfunction or injury. Furthermore, we determined that 55% (10 out of 18) of patients met criteria for Score 0 hepatic GVHD (rising serum ALT <3X ULN without liver biopsy or clear non-GVHD cause) and had documented follow-up surveillance or additional immune suppression [17]. Fortunately, none of these patients with steatosis developed the veno-occlusive disease (clinical or radiologic) or died from hepatic GVHD. Nonetheless, these findings suggest that improved measurements of liver metabolic health (including steatosis) could serve as potential biomarkers for future transplant prognostication models, drug dose adjustments, and metabolism-modulating clinical trials.

### 4.5. Screening for Steatosis

While the gold standard for the diagnosis of steatotic liver disease is biopsy, it is generally not performed in clinical practice due to its invasive nature and associated risks. Hence, CT imaging has been emerging as a valuable screening tool, given its convenience and availability [43]. FibroScan (hepatic elastography) is an ultrasound method that has more recently been shown to measure liver stiffness and steatosis, while avoiding the need for radiation-based imaging like CT. While some studies have demonstrated its utility in predicting post-transplant toxicities, to the best of our knowledge, it is not yet widely adopted into clinical practice.

In our study, we identified 18 out of 306 patients (5.8%) who met the criteria for steatotic liver disease. Prior studies indicate that steatotic liver disease can be noted in close to 70–80% of patients with obesity [44]. Simultaneously, the NHANES real-world study also reported steatotic liver disease rates of 21–27% (by sonography) despite having a BMI less than 30 [45]. There could be several reasons for these discrepancies. The diagnostic sensitivities of contrast-enhanced and non-contrast CT are around 66% and 72%, respectively [46]. Patient selection and lifestyle choices across different geographies may also contribute to the substantial differences in reported steatosis prevalence.

Nonetheless, future studies should consider longitudinal post-transplant measures with a sensitive radiologic study to document the likely progression, regression, and emergence of steatosis and its long-term implications for transplant survivors.

### 4.6. Limitations

With a small cohort of 18 patients identified with hepatic steatosis, reflecting a mixture of underlying malignancies and prior lines of treatment, our study is substantially limited in its ability to evaluate any statistical effect of hepatic steatosis on key outcomes including relapse and mortality. As a descriptive study, our study illustrates the post-transplant course of patients with SLD in our single center, with overall neutral results for patients with a perceived higher-risk co-morbidity. Future higher-powered studies are planned to further evaluate the impact of SLD on allo-HSCT outcomes.

## 5. Conclusions

Overall, our case series suggests allogeneic hematopoietic stem cell transplantation is feasible amongst patients with hematological malignancy impacted with concomitant steatotic liver disease. Nonetheless, given the small size and descriptive nature of our study, larger confirmatory studies will be necessary and much remains to be explored to enhance our understanding of the interplay between hepatic health and immune modulation. Sensitive radiologic tools combined with metabolomics hold promise for future biomarker-driven research in predicting immune perturbation in the context of allo-HSCT.

## Figures and Tables

**Table 1:** Pre-transplant patient characteristics.

Patient Characteristic		
Age at transplant (years)	Median (min–max; N)	56 (30–79; N = 18)
Sex	Female (N)	11
	Male (N)	7
Weight at transplant (kg)	Median (min–max; N)	89.1 (49.6–119; N = 18)
Height at transplant (m)	Median (min–max; N)	1.645 (1.52–1.84; N = 18)
BMI	Median (min–max; N)	33.4 (19.8–46.5; N = 18)
Liver–Spleen Differential (-HU)	Median (min–max; N)	18.8 (10.5–90.0; N = 18)
Race	Caucasian (N)	14
	African American (N)	2
	Hispanic (N)	2
Disease	Lymphomas (N)	2
	MDS/MPN/Other (N)	3
	Acute Leukemias (N)	13
Conditioning Class	Myeloablative (N)	7
	Non-myeloablative (N)	11
Cell Type	Peripheral Blood (N)	17
Donor Type	Related (N)	6
	Unrelated (N)	12
HLA Match’	Well Matched (N)	13
	Partially Matched (N)	4
	Mismatched (N)	1
Post-Transplant Cyclophosphamide	(N)	2
Anti-Thymocyte Globulin	(N)	1
Karnofsky Performance Status	>/=80 (N)	11
	<80 (N)	7
HCT-CI	<=3 (N)	10
	>3 (N)	8
Type II Diabetes	(N)	4
Hyperlipidemia	(N)	7
Hypertension	(N)	8
Cardiovascular Disease	(N)	2
AST	Median (min–max; N)	28 (17–50; N = 18)
ALT	Median (min–max; N)	37 (5–89; N = 18)
Alkaline Phosphatase	Median (min–max; N)	88.5 (61–135; N = 18)
Bilirubin (Total)	Median (min–max; N)	0.305 (0.2–0.84; N = 18)
Albumin	Median (min–max; N)	4.2 (2.6–4.7; N = 18)
Hemoglobin A1c	Median (min–max; N)	6.4 (5.1–9.4; N = 11)

**Table 2:** Clinical outcomes following allo-HSCT in patients with steatotic liver disease.

Patient	Diagnosis	Vital Status	Follow-Up (Months)	Relapse	Time to Relapse (Months)	aGVHD (0-IV)	cGVHD (None/Mild/Mod/Sev)	Cause of Death
1	T-PLL	Dead	11.2	Y	8.3	Grade I	Severe	Relapsed T-PLL
2	MDS	Dead	38.3	Y	9.1	Grade II	None	Relapsed MM
3	ALL	Alive	132.4	N		Grade 0	Moderate/Severe	-
4	AML	Dead	7.9	Y	6.0	Grade 0	None	Relapsed AML
5	ALL	Alive	113.0	N		Grade 0	None	-
6	AML	Dead	16.0	Y	4.5	Grade III	Moderate	Relapsed AML
7	Myeloid sarcoma	Dead	5.6	Y	3.4	Grade 0	None	Relapsed AML
8	AML	Alive	69.9	N		Grade I	Moderate	-
9	AML	Alive	90.5	N		Grade III	Moderate	-
10	AML	Alive	88.9	N		Grade 0	None	-
11	ALL	Alive	97.9	N		Grade 0	Mild	-
12	CTCL	Dead	2.5	Y	0.4	Grade I	None	Relapsed CTCL
13	ALL	Alive	93.1	N		Grade 0	None	-
14	AML	Alive	88.3	N		Grade 0	Moderate	-
15	AML	Dead	5.3	N		Grade 0	Moderate	Infection (NRM)
16	MF	Dead	23.6	Y	7.6	Grade 0	None	Infection (NRM)
17	T-ALL	Dead	34.1	Y	16.7	Grade 0	Severe	Relapsed T-ALL
18	AML	Alive	67.6	N		Grade 0	Severe	-

## Data Availability

The data presented in this study are available on request from the corresponding author due to privacy and legal issues.

## Abbreviations

The following abbreviations are used in this manuscript:

SLD	Steatotic liver disease
MASLD	Metabolic dysfunction associated steatotic liver disease
Allo-HSCT	Allogeneic hematopoietic stem cell transplantation
GVHD	Graft-versus-host disease
